# Psychosocial Impacts of Prophylactic Total Gastrectomy for Hereditary Diffuse Gastric Cancer: A Narrative Review

**DOI:** 10.1002/pon.70304

**Published:** 2025-10-23

**Authors:** Olivia Kearns, Deborah Snyder, Jeremy Davis, Amber F. Gallanis, Rachael Lopez, Michael Liu, Haniya Raza

**Affiliations:** ^1^ Intramural Research Program at the National Institute of Mental Health National Institutes of Health Bethesda Maryland USA; ^2^ University of Maryland Medical System Baltimore Maryland USA; ^3^ Intramural Research Program at the National Cancer Institute National Institutes of Health Bethesda Maryland USA; ^4^ National Institutes of Health Clinical Center Nutrition Department Bethesda Maryland USA

**Keywords:** hereditary diffuse gastric cancer (HDGC), mental health, prophylactic surgery, quality of life

## Abstract

**Background:**

Pathogenic or likely pathogenic (P/LP) *CDH1* germline genetic variants are causally linked to early onset diffuse gastric cancer and prophylactic total gastrectomy (PTG) is a recommended intervention for (P/LP) *CDH1* variant carriers. However, PTG introduces fundamental challenges to a patient's quality of life (QOL) and psychological well‐being. There is a growing need for evidence‐based recommendations to address the psychosocial needs of PTG patients. The present review summarizes published literature on psychosocial sequelae and quality of life reported post‐PTG in germline (P/LP) *CDH1* variant carriers.

**Methods:**

Articles were extracted from the PubMed database using the following search terms: “total gastrectomy”, “CDH1”, “CTNNA1”, “guidelines”, “psychological”, “psychiatric”, “mental health”, “quality of life”, and “longitudinal”. Articles that address psychological and QOL implications and outcomes for PTG or total gastrectomy (TG) patients were included. Articles that mentioned “psychosocial factors” generally, without specific details, were excluded.

**Results:**

37 articles met inclusion criteria. Seven of the most frequently mentioned psychosocial domains in the published literature include quality of life (*n* = 22), social relationships (*n* = 12), depression and anxiety (*n* = 8), decision‐making (*n* = 7), employment and finances (*n* = 4), body image (*n* = 5), and substance abuse (*n* = 3).

**Conclusions:**

Considering the known psychological impact of a genetic cancer predisposition diagnosis and the significant postoperative lifestyle adjustment, this review identifies a need for standardized integration of mental health care along the PTG surgical continuum. The combination of the diagnosis of a hereditary cancer syndrome and the life‐altering nature of prophylactic total gastrectomy (PTG) presents a psychosocial vulnerability for *CDH1* (P/LP) variant carriers.

## 1 Background

Pathogenic or likely pathogenic (P/LP) *CDH1* and *CTNNA1* germline genetic variants are causally linked to early onset diffuse gastric and lobular breast cancer (LBC) [1, 2]. With regard to gastric cancer risk, patients with these variants have two primary risk management options: endoscopic surveillance or prophylactic total gastrectomy (PTG). Increased access to genetic testing has led to more frequent PTG operations. These operations are associated with significant, life‐altering changes to patients' daily lives that can impact psychosocial adjustment. Behavioral changes include eating small but frequent nutrient‐dense meals, avoiding added sugars, modifying physical activity and positioning to prevent post‐gastrectomy symptoms and to preserve lean body mass, and lifelong micronutrition supplementation [3]. Failure to adhere to these changes can result in serious adverse health consequences, including severe unintentional weight loss, metabolic bone disease, hypoglycemia, dehydration, and micronutrient deficiencies [4]. Oftentimes, postoperative discomfort such as bile reflux and dumping syndrome worsen with time from surgery, can persist longitudinally, and may hinder activities of daily living. Collectively, medical complications and the required daily long term behavioral changes can negatively impact patients' mental health and psychosocial adaptation [5].

The most recent International Gastric Linkage Consortium (IGCLC) Guidelines highlight the importance of consulting a psychologist as needed “at routine follow‐up” after PTG; however, recommendations for incorporating mental health clinicians are vague [6]. Conversely, the clinical practice guidelines for general bariatric procedures include a requirement for preoperative psychological screening and detailed recommendations for optimal postoperative follow‐up [7]. In addition to the nutritional and physical life‐altering changes that are secondary to PTG, *CDH1* (P/LP) variant carriers may experience a unique psychosocial burden brought on by the identification of a cancer risk predisposition and the cancer risk‐management decision‐making process. The psychosocial impacts of the diagnosis of a genetic cancer predisposition alone are well‐understood, as these diagnoses can impart burdensome responsibilities and anticipatory distress surrounding decision‐making and one's future risk for disease [8]. Therefore, it is important to consider both practice guidelines from bariatric surgery and research on the psychosocial impact of hereditary cancer syndromes to inform clinical practice and optimize patient outcomes following PTG.

In this review, we summarize the psychosocial sequelae and quality of life (QOL) reported to date post‐PTG in germline *CDH1*(P/LP) variant carriers. We also identify gaps in the current understanding of these psychosocial sequelae and provide recommendations to improve the clinical management of patients with *CDH1* (P/LP) variants who undergo PTG.

## 2 Methods

Papers that were published before May 2025 and written in English were extracted from the PubMed database. Search terms included, “total gastrectomy”, “CDH1”, “CTNNA1”, “guidelines”, “psychological”, “psychiatric”, “mental health”, “quality of life”, and “longitudinal”. Empirical and nonempirical research papers, review papers, book chapters, and editorials were reviewed and determined to be eligible for inclusion. Articles that address psychological and quality of life implications and outcomes for patients who were undergoing or who had previously undergone PTG, or total gastrectomy (TG) were included. Exclusion criteria included research with patients who underwent partial gastrectomy or endoscopic surveillance only and papers that mentioned psychosocial factors generally with non‐specific details and without elaboration. Inclusion and exclusion criteria were decided on by consensus with a psychiatrist, a clinical social worker with expertise in this population, and a research assistant. Each paper was reviewed by the research assistant and summarized into different categories of key information (e.g., sample size, study aims). Further, each paper was organized by the domains it reported on (e.g., depression, quality of life, body image, etc.). This study employed a narrative review methodology, therefore no formal statistical analyses were conducted.

## 3 Results

**FIGURE 1:**
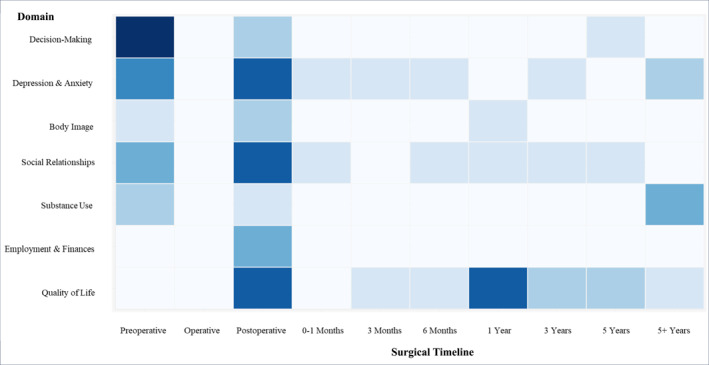
Heatmap saturation of published psychosocial domain research at timepoints along PTG surgical timeline. Darker colors indicate ≥ 5 articles, lightest color indicates 0 articles.

The initial search produced 132 articles dating from 1999 to 2025 from the PubMed database; 37 met inclusion criteria and were used as the basis for this review (Table 1, Table 2, Figure 1). Each article was reviewed for commonly mentioned psychosocial themes. Seven of the most frequently mentioned psychosocial domains in the published literature on germline *CDH1* (P/LP) variant carriers after PTG include quality of life (*n* = 22), social relationships (*n* = 12), depression and anxiety (*n* = 8), decision‐making (*n* = 7), employment and finances (*n* = 4), body image (*n* = 5), and substance abuse (*n* = 3).

**TABLE 1:** Flowchart of identification, screening, and inclusion of articles.

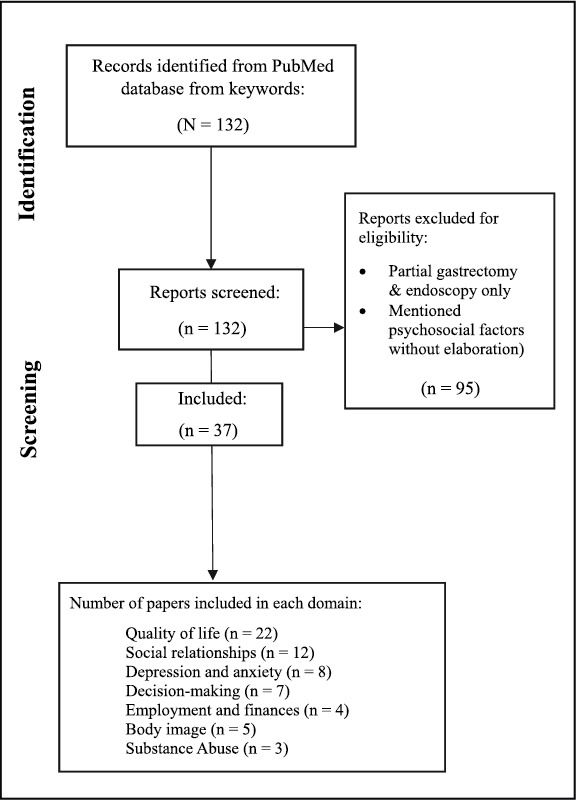

*Note:* Papers could address multiple domains, so the totals of each domain will add up to more than total included articles.

**TABLE 2:** Articles included in review.

Year & authors	Title	Sample	Method	Domain
Ishihara, 1999	Long‐term quality of life in patients after total gastrectomy	*N* = 51	Cross‐sectional survey	Quality of life, employment and finances
Tyrväinen et al., 2008	Quality of life in the long‐term survivors after total gastrectomy for gastric carcinoma	*N* = 25	Cross‐sectional observation	Quality of life, social relationships
Lynch et al., 2008	Hereditary diffuse gastric cancer: Diagnosis, genetic counseling, and prophylactic total gastrectomy	*N* = 17	Retrospective case series	Social relationships
Schrader & Huntsman, 2010	Hereditary diffuse gastric cancer	*N/A*	Narrative review	Quality of life
Chen et al., 2011	A prospective study of total gastrectomy for *CDH1‐*Positive hereditary diffuse gastric cancer	*N* = 18	Prospective cohort	Quality of life
Lynch, Aldoss, & Lynch 2011	The identification and management of hereditary diffuse gastric cancer in a large jordanian family	*N* = 1	Observational case study	Quality of life
Worster et al., 2014	The impact of prophylactic total gastrectomy on health‐related quality of life: A prospective cohort study	*N* = 60	Prospective cohort	Quality of life, body image
Corso et al., 2014	E‐cadherin germline mutation carriers: Clinical management and genetic implications	*N/A*	Review	Quality of life
van der Post et al., 2015	Hereditary diffuse gastric cancer: Updated clinical guidelines with an emphasis on germline CDH1 mutation carriers	*N/A*	Clinical guidelines	Social relationships, depression and anxiety, decision‐making
Takiguchi et al., 2015	Long‐term quality‐of‐life comparison of total gastrectomy by postgastrectomy syndrome assessment scale (PGSAS‐45): A Nationwide multi‐institutional study	*N* = 586	Cross‐sectional, observational cohort	Quality of life
Hallowell et al., 2016	An investigation of the factors effecting high‐risk individuals' decision‐making about prophylactic total gastrectomy and surveillance for hereditary diffuse gastric cancer (HDGC)	*N* = 35	Qualitative phenomenological	Decision‐making
Lee et al., 2016	Postoperative quality of life after total gastrectomy compared with partial gastrectomy: Longitudinal evaluation by European organization for research and treatment of Cancer‐OG25 and STO22	*N* = 182	Prospective cohort	Quality of life, depression and anxiety, body image
Lee et al., 2016	Long‐term quality of life after distal subtotal and total gastrectomy symptom‐ and behavior‐oriented consequences	*N* = 77	Retrospective cohort	Quality of life, social relationships
Hallowell et al., 2016	The psychosocial impact of undergoing prophylactic total gastrectomy (PTG) to manage the risk of hereditary diffuse gastric cancer (HDGC)	*N* = 27	Qualitative interview	Quality of life, employment and finances
Muir et al., 2016	Prophylactic total gastrectomy: A prospective cohort study of long‐term impact on quality of life	*N* = 13	Prospective cohort	Quality of life, depression and anxiety
Strong et al., 2017	Total gastrectomy for hereditary diffuse gastric cancer at a single center: Postsurgical outcomes in 41 patients	*N* = 41	Retrospective cohort	Quality of life, social relationships
Carillo & Santamaría, 2019	Life after a gastrectomy: Experience of patients with gastric cancer	*N* = 17	Qualitative phenomenological	Employment and finances, body image
Drake et al., 2019	Establishing a center of excellence for hereditary diffuse gastric cancer syndrome	*N/A*	Editorial	Quality of life
Kaurah et al., 2019	Hereditary diffuse gastric cancer: Cancer risk and the personal cost of preventive surgery	*N* = 53	Retrospective cross‐sectional	Depression and anxiety
Laszkowska et al., 2020	Optimal timing of total gastrectomy to prevent diffuse gastric cancer in individuals with pathogenic variants in CDH1	*N/A*	Decision‐analytic modeling	Quality of life
Blair et al., 2020	Hereditary diffuse gastric cancer: Updated clinical practice guidelines	*N/A*	Clinical practice guidelines	Quality of life, depression and anxiety, body image, substance abuse
Gamble, Heller, & Davis, 2021	Hereditary diffuse gastric cancer syndrome and the role of CDH1: A review	*N/A*	Narrative review	Substance abuse
McGarragle et al., 2021	Barriers and facilitators to CDH1 carriers contemplating or undergoing prophylactic total gastrectomy	*N* = 24	Mixed methods	Social relationships, decision‐making
Hoskins et al., 2021	Young people's experiences of a CDH1 pathogenic variant: Decision‐making about gastric cancer risk management	*N* = 37	Qualitative semi‐structured interview	Social relationships, depression and anxiety
Liu et al., 2021	CDH1 variants leading to gastric cancer risk management decision‐making experiences in emerging adults: “I am not ready yet”	*N* = 7	Qualitative phenomenological	Social relationships, decision‐making
Forrester et al., 2022	Surgery for hereditary diffuse gastric cancer: Long‐term outcomes	*N* = 43	Prospective cohort	Quality of life
Liu & Lopez, 2022	Emerging adults carrying a CDH1 pathogenic or likely pathogenic variant face diet and lifestyle challenges after total gastrectomy	*N/A*	Narrative review	Social relationships, body image
Wang et al., 2022	Postoperative quality of life after gastrectomy in gastric cancer patients: a Prospective longitudinal observation study	*N* = 117	Prospective cohort	Quality of life
Roberts et al., 2022	International delphi consensus guidelines for follow‐up after prophylactic total gastrectomy: The Life after Prophylactic total gastrectomy (LAP‐TG) study	*N/A*	Delphi consensus guidelines	Quality of life
Stillman et al., 2022	Short and long‐term outcomes of prophylactic total gastrectomy in 54 consecutive individuals with germline pathogenic mutations in the CDH1 gene	*N* = 54	Retrospective cohort	Quality of life
Gamble et al., 2023	Decision‐making and regret in patients with germline CDH1 variants undergoing prophylactic total gastrectomy	*N* = 120	Prospective cohort	Decision‐making
Gallanis & Davis, 2023	Unique challenges of risk‐reducing surgery for hereditary diffuse gastric cancer syndrome: A Narrative review	*N/A*	Narrative review	Decision‐making
Gregory & Davis, 2023	CDH1 and hereditary diffuse gastric cancer: A Narrative review	*N/A*	Narrative review	Social relationships
Gallanis et al., 2023	Costs of cancer prevention: Physical and psychosocial sequelae of risk‐reducing total gastrectomy	*N* = 126	Observational cohort	Quality of life, social relationships, depression and anxiety, employment and finances, substance abuse
Davis & Strong, 2024	Controversies and reasoning in prophylactic total gastrectomy for germline CDH1 pathogenic variant carriers	*N/A*	Narrative review	Quality of life, decision‐making
Şentürk & Korkmaz, 2024	Patients who have undergone total gastrectomy investigation of self‐management experiences on dumping syndrome	*N* = 10	Qualitative interview	Decision‐making
Gallanis et al., 2025	Lessons learned from 150 total gastrectomies for prevention of cancer	*N* = 150	Retrospective cohort	Quality of life, decision‐making

### 3.1 Quality of Life

Twenty‐two of the papers included in this review address quality of life in PTG patients; twelve included empirically derived research studies. Patients' postoperative QOL is impacted by stringent and lifelong daily requirements that can become onerous. Schrader and Huntsman [9] call attention to the necessity of determining the long‐term QOL sequelae of PTG. Drake and colleagues [10] make recommendations for the standardization of multidisciplinary care programs for comprehensive and high‐quality care of hereditary diffuse gastric cancer (HDGC). The authors conclude that QOL should be considered as an important outcome measure in the longitudinal care of patients post‐PTG. A psychologist is recommended to be included in the multidisciplinary care team, highlighting the importance of assessing and intervening around psychosocial variables [11] to optimize QOL outcomes [12].

The studies included in this review use various versions of QOL questionnaires that address physical, psychological, emotional, social, financial, and spiritual domains. Two of the included studies found that QOL scores decreased in the immediate postoperative period but returned to baseline in the longitudinal postoperative period. Specifically, Gallanis and colleagues [13] found that QOL scores decreased one month after gastrectomy, but returned to baseline at the 3‐, 6‐, 12‐, and 24‐month timepoints. Muir and colleagues [14] found that although QOL returned to baseline at the 12‐month mark, scores declined again at 24 months postoperatively. Ishihara [15] found that among 51 PTG patients, 5 rated their post‐operative QOL as good, 15 rated their QOL as fair, 17 rated their QOL as relatively poor, and 14 rated their QOL as poor 2 years or longer postoperatively when adjusted for activities of daily living (i.e., normal activity, restricted activity, sedentary lifestyle, full hospitalization). For many patients, the benefits of gastric cancer risk reduction through PTG outweigh the postoperative consequences. However, some patients experience extremely debilitating surgical sequelae with adverse effects on their QOL [16]. More specifically, long‐term difficulty maintaining adequate oral intake, concerns about body image, and abdominal pain can have negative effects on overall QOL [17].

Two studies compared QOL in patients who underwent partial gastrectomy and patients who underwent PTG. Both studies found that QOL scores were lower in PTG patients when compared to those who underwent subtotal gastrectomies. Specifically, Wang and colleagues [18] reported that patients who underwent distal and pylorus‐preserving gastrectomy had significantly better QOL scores 1 year postoperatively when compared to the PTG patients as measured by the European Organization for Research and Treatment of Cancer (EORTC) Quality of Life Questionnaire—Esophagogastric 25 (EORTC QLQ‐OG25), a cancer‐specific QOL measurement [19]. Lee and colleagues [20] found that patients who underwent subtotal rather than total gastrectomy had higher QOL scores in the domains of social functioning, nausea and vomiting, taste, and eating restrictions as measured by the EORTC Quality of Life Questionnaire ‐ Stomach 22 (EORTC QLQ‐STO22) [21]. This study also found that poorer QOL scores in PTG patients persisted up to 5 years postoperatively, as patients continuously reported symptoms and dietary limitations. The researchers note that beyond the 5‐year postoperative timepoint, most differences in the QOL scores of PTG patients were reduced. However, the eating restrictions domain in the QOL scale continued to reveal lower scores, even beyond 5 years postoperatively [20]. Similarly, J.H. Lee and colleagues [22] found significantly worse postoperative EORTC QLQ‐STO22 and QLQ‐OG25 scores among a group of total gastrectomy patients when compared to a group of partial gastrectomy patients. Continued nutritional and dietary counseling postoperatively can lead to more positive QOL outcomes [5, 23].

Seven studies included in this review describe that PTG patients have positive outcomes and/or do not experience significant worsening of QOL postoperatively. Eleven out of 20 patients assessed for QOL 5 years postoperatively rated their QOL as either “excellent” (*n* = 3) or “very good” (*n* = 8) on the Centers for Disease Control and Prevention Health‐Related QOL Instrument [24, 25]. Additionally, two studies showed that long‐term quality of life following total gastrectomy was acceptable, but researchers did not specify which measures were used to assess QOL [26, 27]. One study concluded that daily life satisfaction in both PTG patients and partial gastrectomy patients is largely unchanged postoperatively. In both groups, the various postoperative sequelae did not majorly impact adjustment or adaptation [28]. Strong and colleagues [29] evaluated long‐term effects of PTG among 41 patients who underwent total gastrectomy for *CDH1* (P/LP) mutation. Postoperative QOL data was collected from 20 patients and compared with a cohort of 35 patients who underwent PTG for nonhereditary gastric cancer. Eighty‐five percent of the CDH1 (P/LP) group reported that their overall postoperative QOL was better or as expected. Thirty percent reported they had no QOL expectations or that their QOL was worse than expected. Tyrväinen and colleagues [30] found no difference in postoperative QOL measures when comparing a sample of total gastrectomy patients and normal population controls. Lynch, Aldoss, and Lynch [31] conducted genetic testing on 23 Jordanian individuals in a large single‐family system, resulting in 13 individuals who were positive for the pathogenic *CDH1* variant. One family member who underwent PTG described her postoperative experience: “…I have survived the surgery and gone back to my active healthy life. It will be very easy for my 2 young [CDH1 mutation carrier] children and others to see that there is good quality of life after gastrectomy” [31].

QOL considerations are integral in the cancer risk management counseling and decision‐making process. Laszkowska and colleagues [32] conducted a study to analyze the long‐term impacts of PTG depending on age at the time of surgery. They conclude that while lower cancer mortality rates are associated with surgery at a younger age, post‐operative QOL may be worse in this population. Authors recommend that there may be ages to undergo surgery that are associated with better postoperative outcomes when considering a balance of both cancer risk management and QOL [32].

### 3.2 Social Relationships

The impact of PTG on social relationships was addressed in 12 of the 37 papers examined. The 2015 IGCLC recognizes family‐related issues as one of the six main problems identified by individuals undergoing genetic counseling and testing for hereditary cancer syndromes. Specifically, individuals reported familial communication problems and responsibility for other family members as predominant problem areas that the guidelines deem necessary to address [33]. A literature review on the current understanding of the genetic role of *CDH1* (P/LP) variants in HDGC revealed that maintaining a family or peer support network is important for postoperative recovery [34]. Further, Strong and colleagues [29] conducted a prospective evaluation of *CDH1* (P/LP) variant carriers from 2005 to 2015 to address pathologic outcomes, postoperative complications, and long‐term QOL effects of PTG. Authors recommend the involvement of a psychosocial support evaluation for *CDH1* (P/LP) carriers being considered for surgery.

Lynch and colleagues [35] conducted an analysis of four families with HDGC to evaluate the decision processes that *CDH1* (P/LP) variant carriers face, noting that education about their genetic predisposition and further counseling may be beneficial. Researchers described the psychological and social support patients felt from their family as a vital part of their decision‐making process to undergo PTG. They further explain that familial solidarity and connectedness were quite prevalent among this cohort [35]. Familial concerns may be a decisional barrier that leads to either delaying or declining PTG altogether, including disruption to family life, navigating single parenthood while recovering postoperatively, and other related caregiver responsibilities [36].

Young adults living with a *CDH1* (P/LP) variant often engage with family members and advocacy groups throughout their experience for support and guidance in their cancer risk management decision‐making process. Additionally, young adults' perceived risk and consequent decision were greatly influenced by their family's history of cancer and experiences postoperatively [37]. Romantic partnerships and social experiences may complicate the decision‐making process for young people (18–39 years old) with a *CDH1* (P/LP) variant [38]. The most recent IGCLC guidelines acknowledge that associated digestive system changes may interfere with intimacy in relationships [6].

Gallanis and colleagues [13] found that social well‐being scores (as characterized by support from friends and family, satisfaction in one's relationships, etc.) significantly decreased in the first month after surgery but returned to baseline by 6 months postoperatively, as measured by the Functional Assessment of Cancer Therapy‐General (FACT‐G) [39] and Gastric (FACT‐Ga) [40], questionnaires used to assess QOL after PTG. Conversely, scores in social functioning were significantly worse 5 years postoperatively among PTG patients when compared to patients who underwent distal subtotal gastrectomy as measured by the EORTC QLQ‐C30 [20]. Further, in discussing the significant diet and lifestyle changes after PTG, Liu & Lopez [3] note that body image concerns among young adults aged 18–29 years old, as they relate to romantic relationships, may develop or become emphasized during these years of age.

In contrast, Tyrväinen and colleagues [30] found that social functioning (as defined by the level of interference of physical or emotional health in normal social activities) in 25 PTG patients was not significantly different when compared to non‐PTG patient controls. The authors further concluded that while physical post‐PTG symptomatology is common in their sample, patients' functioning as it relates to physical, social, and mental health did not differ from the non‐PTG patient controls.

### 3.3 Depression and Anxiety

Eight of the 37 papers included in this review addressed depression, anxiety, and emotional distress. The 2020 HDGC Clinical Practice Guidelines recommend clinicians avoid PTG in patients with psychiatric diagnoses that are refractory to treatment and that cause impairments in daily life, including severe depression [6]. Additionally, the 2015 Clinical Practice Guidelines identified anxiety as one of the 6 dominant problems reported by individuals undergoing genetic counseling and testing for hereditary gastric cancer syndromes [33]. Though these responses were recorded prior to deciding and/or undergoing PTG, the processes of genetic testing and counseling are important to note. Among a group of young people aged 18–39 years old with a germline *CDH1* (P/LP) variant, Hoskins and colleagues [38] found that individuals elected to be scheduled for PTG in part due to the anxiety they experienced in the past when waiting for endoscopy results.

Lee and colleagues [22] conducted a study evaluating the accuracy of specific measures tailored to assess aspects of QOL in 182 PTG patients. At the 3‐month postoperative evaluation, patients who underwent PTG reported worse scores in anxiety as measured by the EORTC QLQ‐OG25 and EORTC QLQ‐STO22. Among 126 individuals undergoing PTG, emotional well‐being scores significantly decreased in the first month after surgery, then returned to baseline by six months postoperatively, as measured by The National Institutes of Health Healing Experience of All Life Stressors (NIH‐HEALS) [41]. Additionally, five patients reported a new diagnosis of depression following their surgery. The researchers note that the QOL questionnaires did not adequately capture many of the challenges patients described in the months after surgery [13]. Ishihara [15] investigated QOL in patients 2 years and beyond postoperatively to assess how nursing care could be more tailored to these individuals' needs. They found that in 51 patients, 8% reported being “constantly anxious,” about their health and recurrence of disease, 47% reported being “sometimes anxious,” and 45% were “not anxious.”

In an investigation of physical and psychosocial outcomes in 18 PTG patients, most patients reported feeling anxiety about their health preoperatively, as measured by the EORTC QLQ‐STO22. Postoperatively, a majority of the sample reported few symptoms of psychological distress as measured by the Brief Symptom Inventory‐18 [14]. Kaurah and colleagues [42] found that in a sample of 53 individuals who underwent PTG, most did not report experiencing general anxiety or depression as measured by the PROMIS and EORTC Short Form 36v.II instruments. Researchers concluded that patients' psychosocial functioning postoperatively was comparable to levels among the general population.

### 3.4 Decision‐Making

Seven of the papers included in this review address the decision‐making process in electing to undergo PTG. There are several factors that influence the decision for *CDH1* P/LP variant carriers to undergo PTG. Reasons for electing to have the procedure may include family history of gastric cancer, low efficacy rates of endoscopic surveillance in detecting cancer, anxiety while awaiting biopsy results, identification of signet ring cells on surveillance, strong social support, personal values and priorities, and trust in healthcare providers [5, 16, 23, 36, 37]. Decisional counseling is important to include in the patient's preoperative course of care (van der Post et al., 2015) [33]. For carriers that decide not to undergo PTG, reasons may include confidence in routine screening, knowing someone with negative post‐operative experience after PTG, familial/work responsibilities, age, and fertility‐related concerns [36]. Decision regret among patients who underwent PTG is associated with postoperative complications and lack of evidence of cancer on final gastrectomy specimen [4, 43, 44].

### 3.5 Employment and Finances

Four papers included in this review are research studies that address employment and finances. Among a sample of 68 patients who underwent PTG, 23% reported an employment change post‐operatively [13]. Changes to occupation were attributed to the inability to perform work tasks, chronic fatigue, persistent gastrointestinal symptoms including nausea, and/or the inability to eat frequent meals while working. Two patients reportedly changed jobs to have access to better medical care [13]. Ishihara [15] reported that out of 38 patients who were employed preoperatively, 13 resumed work as normal, 3 had to take leave, 5 had to reduce their workload/change their job, and 17 retired (5 of these due to age).

In qualitative interviews with 27 postoperative PTG patients, clinicians reported that a significant group of interviewees from a Familial Gastric Cancer Study in the UK described experiences of negative financial consequences postoperatively including medical debt, inability to access welfare during extended leave, or no longer being able to work full‐time due to symptomatology, especially for those working manual occupations [45].

“Wanting to feel useful” was a central issue for some postoperative PTG patients [46]. Those who were once the primary source of income for their families report familial conflicts resulting from taking leave from work, changing occupations, and other employment‐related troubles. Researchers note that more attention should be given to the burden of financial worries due to the longitudinal medical care and the nutrition requirements associated with PTG that are often expensive [46].

### 3.6 Body Image

Five papers included in this review addressed body image. Three of these papers are publications on empirical research. The IGCLC Guidelines acknowledge that changes to the digestive system because of PTG may interfere with self‐confidence as it relates to the body [6]. Inevitable postoperative weight loss in young adults (18–29 years) may lead to body image issues, including the role of body image in romantic relationships, physical activities, and self‐identity [3]. Total gastrectomy patients report poorer body image scores postoperatively when compared to partial gastrectomy patients as measured by the EORTC QLQ‐STO22 [22]. Carillo and Santamaria [46] conducted qualitative interviews with 17 patients within the first year following PTG. They determined that body image, including feeling weaker and being more susceptible to weight loss, was a central issue for these patients.

In a prospective, multicenter study conducted to quantify both the physical and psychological effects of PTG, 44% (14/32) of patients had persistent body image issues beyond 12 months postoperatively as measured by the EORTC QLQ‐STO22. The authors note that the questionnaires administered did not adequately capture the full effect of some physical and mental health symptoms post‐PTG. Further qualitative investigation beyond the standardized questionnaires was needed to make accurate conclusions on patients' experiences [17].

### 3.7 Substance Abuse

Three of the 37 papers included in this review addressed substance abuse. The 2020 IGCLC Guidelines state that untreated addictions, including alcohol or tobacco, should be addressed preoperatively and that attention should be given to changes in the effects of alcohol postoperatively [6]. Alcohol metabolism is altered in post‐PTG patients due to rapid transit and absorption, resulting in a sharp increase in blood alcohol content and subsequent risk of acute alcohol intoxication [47]. Additionally, Blair and colleagues recommend clinicians closely monitor the effects of alcohol use on health and psychological outcomes in these patients, citing the increased effects of alcohol [6]. In a retrospective analysis of patients who underwent PTG, one of 126 patients developed alcohol‐use disorder postoperatively and required inpatient rehabilitation [13]. Gamble, Heller & Davis [48] reviewed current topics in the management of HDGC and *CDH1* P/LP variant carriers and formed surgical considerations based on their review and their own clinical experience. They conclude that it is imperative to preoperatively screen for various psychosocial risk factors, including alcohol dependence, to avoid negative outcomes.

## 4 Discussion

The combination of the diagnosis of a hereditary cancer syndrome and the life‐altering nature of prophylactic total gastrectomy presents a unique challenge for *CDH1* (P/LP) variant carriers. Anticipation of a cancer diagnosis that could be prevented with a prophylactic operation that has major longitudinal physical, behavioral, financial, and psychological consequences requires the integration of psychosocial care through the course of patients' medical treatment. This review of extant research found 7 psychosocial domains most impacted by PTG. There was general agreement on the impact of PTG on changes in body image, the importance of social support over the entire perioperative period, postoperative adjustments to employment/financial situations, and the necessity for specific clinical attention to any history of substance use and its risks. In addition, there are studies that identify associations between PTG and depression, anxiety and decreased QOL. The lack of consensus on the impact of PTG on these domains, the significant gaps in descriptions of other psychosocial outcomes longitudinally (see Figure 1), and the lack of agreement in research findings make it challenging to draw definitive conclusions. This highlights a need for further investigation into when and how to integrate mental health support in the treatment of PTG patients. These data could inform the development of future guidelines, patient education and appropriate interventions. The current state of evidence on the impact of PTG on the aforementioned psychosocial domains highlights the need to move beyond vague recommendations about integrated mental health care.

### 4.1 Clinical Implications

For the last 30 years, clinical practice guidelines for bariatric procedures have recommended a formal psychological evaluation before surgery [49]. The 2019 bariatric surgery guidelines specify various psychosocial domains to assess in this preoperative evaluation, including eating‐disorder symptoms, past mental health treatment, cognitive functioning, social support, quality of life, and more. These guidelines also emphasize the importance of including a mental health professional on the multidisciplinary care team and acknowledge aspects of the bariatric surgical course that are improved because of this involvement [7]. Considering the known psychological impact of a genetic cancer predisposition diagnosis, the invasiveness of PTG, and the significant postoperative lifestyle adjustment similar to some bariatric procedures, clinical practice guidelines for *CDH1* (P/LP) variant carriers would benefit from charting a similar course.

*CDH1* (P/LP) variant carriers need a more comprehensive approach to evaluating their biopsychosocial needs to optimize their pre‐ and postoperative adjustment. As an example of the importance of this approach, the 2020 IGCLC Guidelines recommend PTG for carriers who are between 20 and 30 years of age [6]. These 2 decades of life come with significant normative developmental milestones, including higher education, establishing a career, and family planning that risk being negatively impacted by the surgery. Of note, the reproductive decision‐making process in PTG is significantly understudied and needs further investigation. Moreover, PTG patients may also face the dual burden of lobular breast cancer risk. For those patients with LBC, intensive cancer therapy and decisions related to mastectomy may influence choices related to treatment of HDGC and PTG. Research that examines the most effective evaluation methods, namely validated screening tools, clinical gold standard evaluation, or a combination of both, would be useful in identifying those surgical candidates who might be at high risk for psychosocial morbidity.

### 4.2 Limitations

Limitations of this review include potentially missing literature that is available through other databases, as this review was conducted using only the PubMed database. Including only English articles also potentially limits the full yield of articles. Study heterogeneity, lack of objective assessment tools, and mostly small sample sizes also limit our ability to draw comprehensive conclusions.

## 5 Conclusion

This review describes the current state of psychosocial research in germline *CDH1* P/LP variant carriers who elect to undergo PTG for management of inherited diffuse gastric cancer risk. While some undergo PTG without difficulty, a significant number of patients, especially young adults, report that postoperative adjustment can be substantial and can hinder returning to one's preoperative lifestyle, including employment, socializing, intimate relationships, reproduction, and other activities of daily living. We describe the current state of psychosocial research by highlighting 7 psychosocial domains that are impacted by PTG, including quality of life, social relationships, depression and anxiety, decision making, employment and finances, body image, and substance use. Future research can help to drive clinical interventions that standardize psychosocial care and inform clinical guidelines.

While it is recognized that psychosocial evaluation is recommended for *CDH1* P/LP variant carriers undergoing elective PTG, the nature of this involvement has not been clearly defined. Given the well‐established guidelines for bariatric procedures, it is important to consider engaging psychosocial experts for the purpose of integrating a preoperative psychological evaluation [50] with longitudinal follow‐ups, particularly for PTG patients who are identified at high risk. A range of mental health clinicians, including psychologists, social workers, and/or psychiatrists, should be integrated as key members of the larger PTG multidisciplinary team. The development of protocols where patients are proactively screened and managed both pre‐ and post‐operatively for various psychosocial risk factors will likely improve the systematic care of PTG patients and thus optimize patients' overall surgical outcome and quality of life.

## Author Contributions

The contributions of the NIH author(s) were made as part of their official duties as NIH federal employees, are in compliance with agency policy requirements, and are considered Works of the United States Government. However, the findings and conclusions presented in this paper are those of the author(s) and do not necessarily reflect the views of the NIH or the U.S. Department of Health and Human Services.

## Funding

This research was supported by the Intramural Research Program of the National Institutes of Health (NIH) (Annual Report Number ZIAMH002922).

## Conflicts of Interest

Author R.L. is a Commissioned Officer in the United States Public Health Service (PHS). The opinions and assertions expressed herein are those of the authors and are not to be construed as reflecting the views of the PHS, Uniformed Services University of the Health Sciences, or the United States Department of Defense.
